# Theta-burst transcranial magnetic stimulation promotes stroke recovery by vascular protection and neovascularization

**DOI:** 10.7150/thno.51573

**Published:** 2020-10-26

**Authors:** Xuemei Zong, Yuyu Li, Cui Liu, Wenxuan Qi, Dong Han, Lorelei Tucker, Yan Dong, Shuqun Hu, Xianliang Yan, Quanguang Zhang

**Affiliations:** 1 Emergency Medicine Department of the Affiliated Hospital of Xuzhou Medical University, 99 Huaihai Road, Quanshan District, Xuzhou, Jiangsu Province, 221002, China.; 2 Department of Neuroscience and Regenerative Medicine, Medical College of Georgia, Augusta University, 1120 15th Street, Augusta, GA 30912, USA.

**Keywords:** ischemic stroke, Transcranial Magnetic Stimulation (rTMS), vascular protection, angiogenesis, vascular repair, HIF-1α

## Abstract

**Rationale:** The integrity and function of the blood-brain barrier (BBB) is compromised after stroke. The current study was performed to examine potential beneficial effects and underlying mechanisms of repetitive transcranial magnetic stimulation (rTMS) on angiogenesis and vascular protection, function, and repair following stroke, which are largely unknown.

**Methods:** Using a rat photothrombotic (PT) stroke model, continuous theta-burst rTMS was administered once daily to the infarcted hemisphere for 5 min, beginning 3 h after PT stroke. This treatment was applied for 6 days. BBB integrity, blood flow, vascular associated proteins, angiogenesis, integrity of neuronal morphology and structure, and behavioral outcome were measured and analyzed at 6 and/or 22 days after PT stroke.

**Results:** We report that rTMS significantly mitigated BBB permeabilization and preserved important BBB components ZO-1, claudin-5, occludin, and caveolin-1 from PT-induced degradation. Damage to vascular structure, morphology, and perfusion was ameliorated by rTMS, resulting in improved local tissue oxygenation. This was accompanied with robust protection of critical vascular components and upregulation of regulatory factors. A complex cytokine response was induced by PT, particularly at the late phase. Application of rTMS modulated this response, ameliorating levels of cytokines related to peripheral immune cell infiltration. Further investigation revealed that rTMS promoted and sustained post-ischemic angiogenesis long-term and reduced apoptosis of newborn and existing vascular endothelial cells. Application of rTMS also inhibited PT-induced excessive astrocyte-vasculature interactions and stimulated an A1 to A2 shift in vessel-associated astrocytes. Mechanistic studies revealed that rTMS dramatically increased levels of PDGFRβ associated with A2 astrocytes and their adjacent vasculature. As well, A2 astrocytes displayed marked amplification of the angiogenesis-related factors VEGF and TGFβ. PT induced a rise in vessel-associated expression of HIF-1α that was starkly intensified by rTMS treatment. Finally, rTMS preserved neuronal morphology, synaptic structure integrity and behavioral outcome.

**Conclusions:** These results indicate that rTMS can exert powerful protective and restorative effects on the peri-infarct microvasculature after PT stroke by, in part, promoting HIF-1α signaling and shifting vessel-associated astrocytic polarization to the A2 phenotype. This study provides further support for the potent protective effects of rTMS in the context of ischemic stroke, and these findings implicate vascular repair and protection as an important underlying phenomenon.

## Introduction

Ischemic stroke, or focal cerebral ischemia, is a leading cause of disability and death worldwide, impacting nearly 800,000 each year in the US, leaving survivors with profound motor and cognitive disability 1. Ischemic stroke commences when blood flow is blocked or reduced to portions of the brain and leads to cell death if reperfusion does not occur in a narrow time window. This region, the core infarct, is surrounded by the penumbra, a region of tissue that will develop into infarcted tissue if intervention does not occur. Because of this, the penumbra is a valuable target for stroke therapies 2-6. Unfortunately, patients are limited to only two approved treatment options, mechanical thrombectomy and thrombolysis through tPA administration 3, 7. This deficit of available options necessitates novel methods by which the damage of stroke can be abated or reversed.

The brain is an immune-privileged region of the body, cordoned off from the rest of the body via the blood brain barrier (BBB). The BBB is a complex structure, comprised of pericytes, endothelial cells, and astrocytic endfeet, held together firmly with tight junction proteins like claudins and occludins, that allow for fine-tuned regulation of influx and efflux of compounds across the cerebral vasculature 8, 9. Through this structure, the neurovascular unit, astrocytes are able to regulate blood flow in conjunction with neuronal activity to match metabolic needs 9. After infarct, the integrity of this structure is compromised 10. This causes dysregulation of solute transport and allows for infiltration of peripheral immune cells, driven by the release of inflammatory cytokines 10-12. While this immune response may be beneficial in the early stages after stroke, prolonged disruption of the BBB and the subsequent neuroinflammatory response is detrimental to reparative processes 4, 12. Several studies in experimental animal models of stroke positively associate BBB integrity with improved histological and neurological outcomes 13-16. Integrity of the vasculature and revascularization of the peri-infarct region can potentially support recovery long-term.

Revascularization is driven by the process of angiogenesis, wherein new vessels are generated from existing vasculature 17. Angiogenesis is initiated early after ischemic insult by the release of angiogenic factors like platelet-derived grow factor receptor beta (PDGFRβ), vascular endothelial growth factor (VEGF), and insulin-like growth factor binding proteins (IGFBPs) by cells in a response to exposure to a hypoxic environment 17-20. Endothelial cells respond to this signal by proliferating and migrating towards the VEGF gradient, degrading the vascular basement membrane with matrix metalloproteinases 17, 21. As well, circulating epithelial progenitor cells migrate towards the region, releasing more stimulating factors that promote further proliferation and migration 17. Within 3 days of injury, the process of angiogenesis is in full swing, with vasculature beginning to form in the peri-infarct area 22, 23. These new vessels, much like nascent neurons formed in response to stroke, do not last long-term in the post-stroke environment 22, 23. It is suggested that this process may be more to support clearance of cellular debris than any long-term rejuvenation efforts 22-25. Despite this, however, angiogenesis is often coupled with neurogenesis, as angiogenic niches often overlap with proliferating neural progenitor cells 22, 23. Therefore, supporting angiogenesis long-term may help improve neurogenesis as well, perhaps boosting restorative processes and improving functional recovery after stroke 22, 23.

Transcranial magnetic stimulation is a non-invasive therapy wherein a magnetic field is applied to a targeted region of the brain, inducing a current in a region of interest 26. At high frequencies, this induced current stimulates activity, while lower frequencies inhibit activity 27. Smaller pilot trials and case studies have shown that repetitive Transcranial Magnetic Stimulation (rTMS) administration can reduce symptoms of various psychiatric disorders like unipolar depression, obsessive-compulsive disorder, and alcohol abuse disorder 28, 29. Several animal studies have also investigated the effects of rTMS on injury and disease models, demonstrating that rTMS can have neuroprotective effects and can improve neurological function 30, 31.

Both glutamatergic and GABAergic neurotransmissions are critically involved in the response of the human motor cortex to theta-burst rTMS 32, 33. Previous studies show that theta-burst rTMS can promote phasic GABA inhibition, thereby promoting stroke recovery. In addition, one such rTMS paradigm has been shown to induce an LTD-like change in plasticity, modulating neuro-cortical excitability via several mechanisms that promote GABAergic inhibition 34, 35. An increasing body of research has been undertaken to evaluate the effects of theta burst TMS on pathophysiological conditions in humans *in vivo*, such as in mental disorders 36-38 and aging-related diseases 39, 40. Our previous work demonstrated that theta-burst rTMS treatment shifted the balance of glial activation to anti-inflammatory phenotypes, reduced oxidative stress, protected against neuronal cell death, and led to improved functional outcomes in the rat PT stroke model 41. Another study shows that theta-burst rTMS can upregulate angiogenesis-related proteins, such as MMP-9 and VEGF 42. Building on these previous reports, this study was conducted to investigate whether rTMS can preserve vascular integrity, promote angiogenesis, and support vascular repair and function after experimental stroke.

## Materials and Methods

### Photothrombotic (PT) Stroke Model and Repetitive Transcranial Magnetic Stimulation (rTMS) Treatment

Three-month-old healthy male Sprague-Dawley rats were randomly assigned to into the following 3 groups: a control group without PT stroke (Ctl group), (b) an experimental group with PT stroke, which received sham rTMS treatment (PT stroke group), and (c) an experimental group with PT stroke, which received rTMS treatment (rTMS group). In some experiments, an additional control group of healthy, non-stroke rats was treated with rTMS in the same manner as stroke animals to determine if rTMS promotes angiogenic activity. Five-min theta-burst rTMS treatment (3 pulses of 50 Hz, repeated every 200 ms) was applied to the animals by targeting the infarcted hemisphere using a pair of Helmholtz coils, as described previously 41. Treatment was initiated 3 h after PT stroke and administered daily from day 1 to day 6 after stroke. The intensity of the magnetic field delivered to the infarcted hemisphere was adjusted to 200 Gauss (FW Bell RoHS meter #5180, OECO LLC, OR, USA). During treatment, the animals were restrained in a transparent DecapiCone positioned between Helmholtz coils, and returned to their home cage thereafter. For sham rTMS stimulation, the procedure was performed identically as with rTMS-treated stroke animals, except that the rTMS magnetic field was adjusted to zero. PT stroke injury model was performed as described previously by our laboratory 41, 43, 44. Briefly, the rats were anesthetized with an intraperitoneal injection of sodium pentobarbital (50 mg/kg body weight), and animals were placed in a stereotactic frame. The scalp was longitudinally incised with a single cut and the skull surface was exposed and cleaned with hydrogen peroxide. A fiber-optic cable guided cold-light beam (150W, KL 1500 electronic, Carl Zeiss, Germany) of 6 mm diameter was stereotactically positioned onto the skull with the light spot centered at 1.8 mm anterior to the bregma and 2.5 mm lateral from the midline. Five min after injection of Rose Bengal dye (0.1 mg/g body weight, i.p.), the skull was illuminated for 15 min. Afterwards, the skin was sutured and rats were returned to their cages. During and after surgical procedures, rectal temperature was monitored and maintained at 36.5-37.5 °C by a thermostatically controlled heating pad. All animal procedures were approved by the Institutional Animal Care and Use Committee (IACUC) of the university and complied with the guidelines of National Institutes of Health. Every effort was made to minimize animal pain and distress the number of animals used in the experiment.

### Regional Cerebral Blood Flow Monitoring

Laser Doppler Flowmetry (LDF) was used to monitor the cerebral blood flow at the skull surface of the infarcted area as reported in our previous study 41. In brief, the animals were fixed in the stereotaxic frame under isoflurane anesthesia at 6 h, 6 d and 22 d following PT stroke. A laser OxyFlo XP Probe connected to an Oxy-Lab LDF Microvascular Perfusion Monitor (Oxford Optronix Ltd, UK), was vertically located at the ischemia-infarcted center. Changes in blood perfusion units (BPU) representing blood flow for each animal were continuously monitored for 5 min, and the values were recorded over the testing period for data analysis. The baseline level of blood perfusion was acquired by monitoring the cerebral blood flow of the subjects without ischemic stroke.

### Brain Perfusion, Tissue Preparation and 3,3'-diaminobenzidine (DAB) Staining

As described in our previous study 45, animals were transcardially flushed with ice-cold saline under deep anesthesia, followed by perfusion of 4% paraformaldehyde (PFA) in 0.1 M phosphate buffer (PB). Brains were removed from the skull, post-fixed overnight with PFA, and cryoprotected with 30% sucrose. Coronal sections (40 µm) along the ischemia-infarcted cortex were collected. DAB staining was performed according to our previous protocol 45. Briefly, sections were blocked with 10% normal goat serum in PBS containing 0.1% Triton X-100 and 0.3% H_2_O_2_ for 1 h at room temperature. Sections were then incubated with the anti-rat IgG or anti-RECA1 primary antibody overnight at 4 °C. After three washes, biotinylated secondary antibody (1:200, Vector Laboratories) was added and incubated for 1 h. Afterwards, sections were washed, followed by incubation with ABC reagents for another 1 h. Sections were then rinsed and incubated with DAB reagent (Vector Laboratories) for 2-10 min. Finally, tissues were washed briefly with distilled water and dehydrated in graded alcohols, cleared in xylene, and mounted using xylene-based mounting medium. Bright-field color images were captured on an Olympus microscope BX60.

### BrdU, Fluorescein isothiocyanate-dextran (FITC-dextran) and Evans blue Injections

In order to examine cell proliferation, BrdU (50 mg/kg body weight) was intraperitoneally administered to each animal once daily over a 6-day period, initiated 3 h after PT stroke. To measure microvascular perfusion, FITC-dextran (2000 kD, 0.1 ml of 50 mg/mL, Sigma-Aldrich) was administered through the tail vein 1 min before sacrifice 46. The brain was rapidly removed, without saline flush, and fixed for 24 h in 4% of PFA at 4°C. Coronal sections were then collected on a vibratome as describe above. To quantify blood-brain barrier permeability, FITC-dextran (70 kD, 0.2 ml of 50 mg/mL, Sigma-Aldrich) and Evans blue (50 mg/kg, Sigma-Aldrich) were injected intravenously through the tail vein simultaneously, and were allowed to circulate in the rat for 4 h. Rats were then transcardially flushed with ice-cold saline followed by PFA perfusion. Brain sections were then cut and subjected to confocal imaging. Evans blue content in the peri-infarct cortical proteins (30 μg each sample) of the saline-flushed brain was also measured on a fluorescence spectrophotometer (Perkin Elmer) with excitation at 620 nm and emission at 680 nm as described 47. BrdU, FITC-dextran, and Evans blue injections were administrated to all groups of animals.

### Local Hypoxia Measurement

To detect local hypoxia and the changes of local reoxygenation status, animals were intravenously injected with pimonidazole hydrochloride (Hypoxyprobe-1, 30 mg/kg in 0.9% saline via tail vein, Hypoxyprobe, Inc.). Animals were sacrificed 1 h after injection of pimonidazole, followed by transcardial flash with saline, PFA perfusion, and brain section preparation as describe above. Pimonidazole is reduced in hypoxic cells to form stable adducts, and the amount detected is proportional to the hypoxic level of the tissue, which can be quantified by pimonidazole immunohistochemistry using anti-pimonidazole antibody supplied in the hypoxyprobe 1 kit.

### Immunofluorescence staining and Confocal Microscopy

As described previously 41, the peri-infarct brain area of coronal slices was chosen for imaging and analysis. Immunofluorescence staining of free-floating sections was performed following our previous study 48. Briefly, tissue sections were washed with 0.4% Triton X-100 for 3 × 10 min, followed by 1 h blocking (10% normal donkey serum), and overnight primary antibody incubation at 4 °C. The following primary antibodies were used: anti-rat IgG (Vector Laboratories); anti-pimonidazole (Hypoxyprobe, Inc.); anti-VEGF (Proteintech); anti-CD31 (Cell Signaling); anti-Ki67, MBP, NeuN, RECA1, BrdU, PDGFRβ, TGFβ, GFAP, C3d and S100A10 (Thermo Fisher Scientific); anti-Synaptophysin and Spinophilin (Abcam); and anti-TGF-β and HIF-1α (Santa Cruz). Sections were then washed three times with PBS-Triton X-100 at room temperature, followed by incubation with appropriate secondary antibodies conjugated to Alexa Fluor (Thermo Fisher Scientific) for 1 h. After washes, the sections were mounted and coverslipped in Vectashield mounting medium without or with DAPI (Vector Laboratories).

For detection of BrdU co-labeled cells, PFA-fixed brain sections were permeabilized sequentially with 1M HCl for 10 min at 0 °C, 1M and 2M HCl for 10 min each at room temperature, then 2M HCl for 20 min at 37 °C, followed by neutralization with 0.1M sodium tetraborate (pH 8.5) for 20 min. Samples were then washed 3 times, blocked, and finally incubated with anti-BrdU antibody combinations overnight at 4 °C.

TUNEL staining was performed on free-floating sections using a Click-iT® Plus TUNEL assay kit (Thermo Fisher Scientific) following the manufacturer's protocol, as detailed in our recent study 41. All the double and triple-stained sections were visualized and captured with a LSM700 Meta confocal laser scanning microscope (Carl Zeiss, Germany) in XYZ plane using a 40 × oil immersion Neofluor objective. The image size was set at 512×512 pixels and scanned using Z-series scanning (20 optical slices) with 12-bit pixel depth using optimum pinhole diameter. The Z-stacks were then converted into three-dimensional (3D) projection images using ZEN imaging software from Carl Zeiss, and representative fluorescent images were presented. In the counting studies of double staining labeled cells (RECA1/Ki67, BrdU/CD31, and RECA1/TUNEL) and triple labeled endothelial cells (RECA1/BrdU/TUNEL), the numbers of co-labeled cells were counted in 3-5 representative capture views of the peri-infarct brain areas from each animal. Cell counts on the examined sections were then averaged to provide a single value for the specific group. A mean ± SE was calculated from the data in each group and used to determine between groups.

### Duolink II Proximity Ligation Assay (PLA)

Duolink II *in situ* PLA immunostaining for ZO-1 and Claudin-5 interactions was performed using the Duolink *in situ* detection reagent kit (Sigma-Aldrich) according to the manufacturer's protocol, as described in our recent work 49. Briefly, brain sections were blocked for 1 h and incubated overnight with mouse and rabbit primary antibodies at 4 °C overnight. After 3 washes, the sections were incubated with Duolink PLA Rabbit MINUS and PLA Mouse PLUS proximity secondary probes for 1 h at 37 °C. For ligation and circularization, ligation mix was added after washing and incubated for 30 min at 37 °C. Sections were then washed, mounted on the slides, and sealed in mounting medium. PLA signals as distinct dots representing the protein-protein interactions were visualized and captured under a LSM700 confocal microscope. Dot density analyses were performed using Imaris software (Bitplane Inc.).

### Morphometric Measurement of Vascular and Astrocytic Parameters

Morphometric determinations of the potential changes of vascular and astrocytic parameters, as well as microvessel associated proteins, were performed using the peri-infarct zone 22 days after stroke. Schematic diagrams of techniques and software used are shown in **Figure 3C**. As necessary, vascular structures were stained and visualized with an endothelial marker (RECA-1), using the DAB peroxidise substrate kit as described above. The acquired DAB-labeled images were thresholded, noise filtered, and binarized to separate vascular segments using Fiji software (ImageJ, NIH, MD, version 1.52q). Vascular branch density (% area, vasculature area/total selection area) and feret diameter were analyzed by using the Fiji “Analyze Particles” measurement module. Vascular length density was calculated as the ratio of skeletonized vasculature to total measured area (% area), and the tortuosity index was calculated as the ratio of total branch length to total euclidean distance.

In addition, Z-stacked confocal images of the peri-infarct regions were acquired using a Zeiss LSM 700 confocal microscope. Representative 3D images were reconstructed, followed by surface rendering using Imaris 9.5.0 software (Bitplane, Switzerland). Vascular surface area analysis and vascular volume analysis were then calculated using the Imaris measurement module. Microvessel associated protein density was performed by 3D quantification of the proteins of interest (such as PDGFR-β, VEGF and HIF-1α) within select microvessels from 3D contour surface as described 50. Surface rendering of GFAP along with RECA1 projection was utilized to visualize their interactions. Total astrocytic surface area, vessel associated astrocytic area and vascular astrocytes volume (over 200 mm^3^) were measured using Bitplane Imaris software, respectively. At least 3-5 cortical sections per animal were selected for staining and analysis, and the representative images are shown.

### Western Blotting Analysis

Brain homogenates and protein preparations were carried out as described in our previous study 41. Briefly, peri-infarct brain tissues of PT stroke animals and the control regions of sham controls were quickly dissected from the isolated brain and frozen in liquid nitrogen. Protein concentrations were determined using a Modified Lowry Protein Assay kit (Pierce, Rockford, IL). Western blotting assays were conducted as we previously described 41. Firstly, proteins (30-50 μg) were separated on sodium dodecyl sulfate-poly-acrylamide gel electrophoresis (SDS-PAGE) and transferred to PVDF membrane. The membranes were blocked for 1 h at room temperature using blocking buffer, followed by separate incubation with primary antibody at 4 °C overnight. The following antibodies were used in the immunoblotting experiments: anti-Occludin, Collagen IV (Col IV), IGFBP1, PDGFRβ, TGFβ, HIF-1α (Thermo Fisher Scientific); anti-ZO-1, Claudin-5, Caveolin-1, MPP2 and MPP9 (Santa Cruz), and anti-VEGF, and β-Actin (Proteintech). Dilution and concentration used were optimized according to the antibody protocol instructions. After washes, the membranes were incubated with horseradish peroxidase (HRP)-conjugated secondary antibodies for 1 h at room temperature. Subsequently, chemiluminescent signals were captured with a CCD-based imaging system (ImageQuant LAS 4000, GE Healthcare) and semi-quantitatively analyzed using Fiji/ImageJ software. Relative level of protein expression was determined by the normalization of respective band density to corresponding loading control (β-Actin). Data were present as means ± SE for graphical presentation and statistical analysis.

### Proteome Profiler Rat Cytokine Analysis

Cytokine array analysis was performed with the prepared proteins from peri-infarct cortical region of PT stroke and the sham control animals as we have described 41. The experimental procedures were conducted using Proteome Profiler Rat Cytokine Array Panel A (R&D Systems, Inc., MN) according to the manufacturer's instructions. In brief, the array membrane was blocked with Array Buffer 6, followed by the addition of protein samples from each experimental group (total 800 μg pooled equally from each animal of the same treatment condition). The sample-detection antibody mixture was then loaded on the array's membrane and incubated on a rocking shaker overnight. After 3 washes, streptavidin-HRP solution was added to the membrane and incubated for 30 min at room temperature. Chemi Reagent mix was then added after washing. Cytokines were detected in duplicate and signal dots were captured using a chemiluminescence system as described above. The mean density of each pair of luminescence was normalized to reference spots from the same membrane. Relative level of cytokines was calculated and analyzed with Fiji/ImageJ software.

### Behavioral Assessments

Grip strength test of the contralateral paw was performed before (baseline) and 22 days after PT stroke, as described with minor modifications 51. Briefly, animal was restricted using the index finger of the operator while leaving the animal's right limb unrestrained. The left forelimb was allowed to grasp a spring balance coupled with a Newton meter. Eight measures for the limb were randomly performed. Intermediate values were averaged for each subject after exclusion of the highest and lowest values. For the elevated body swing test, used to evaluate asymmetric motor behavior, lateral movements of animals were examined 22 days after PT stroke by elevating their tails 10 cm above the desktop surface, as previously described 52. The frequency of left or right swings was scored over three sets of 10 consecutive trials. Body swing test scores were calculated and expressed as the percentage numbers of left-biased turns of the upper body over 10°.

### Statistical Analysis

All the data were expressed as mean ± SE and data analysis was carried out by SigmaStat 3.5 software (SPSS, Inc., IL, USA). The statistical significance was determined using one-way analysis of variance (ANOVA) or a repeated measures 2-way ANOVA (grip strength tests) followed by Student-Newman-Keuls test or Dunnett's test for comparison of multiple groups or compare differences with the control group. *P* < 0.05 was considered as statistically significant.

## Results

### rTMS treatment reduced PT stroke induced permeabilization of the BBB in the peri-infarct region

**Figure 1:**
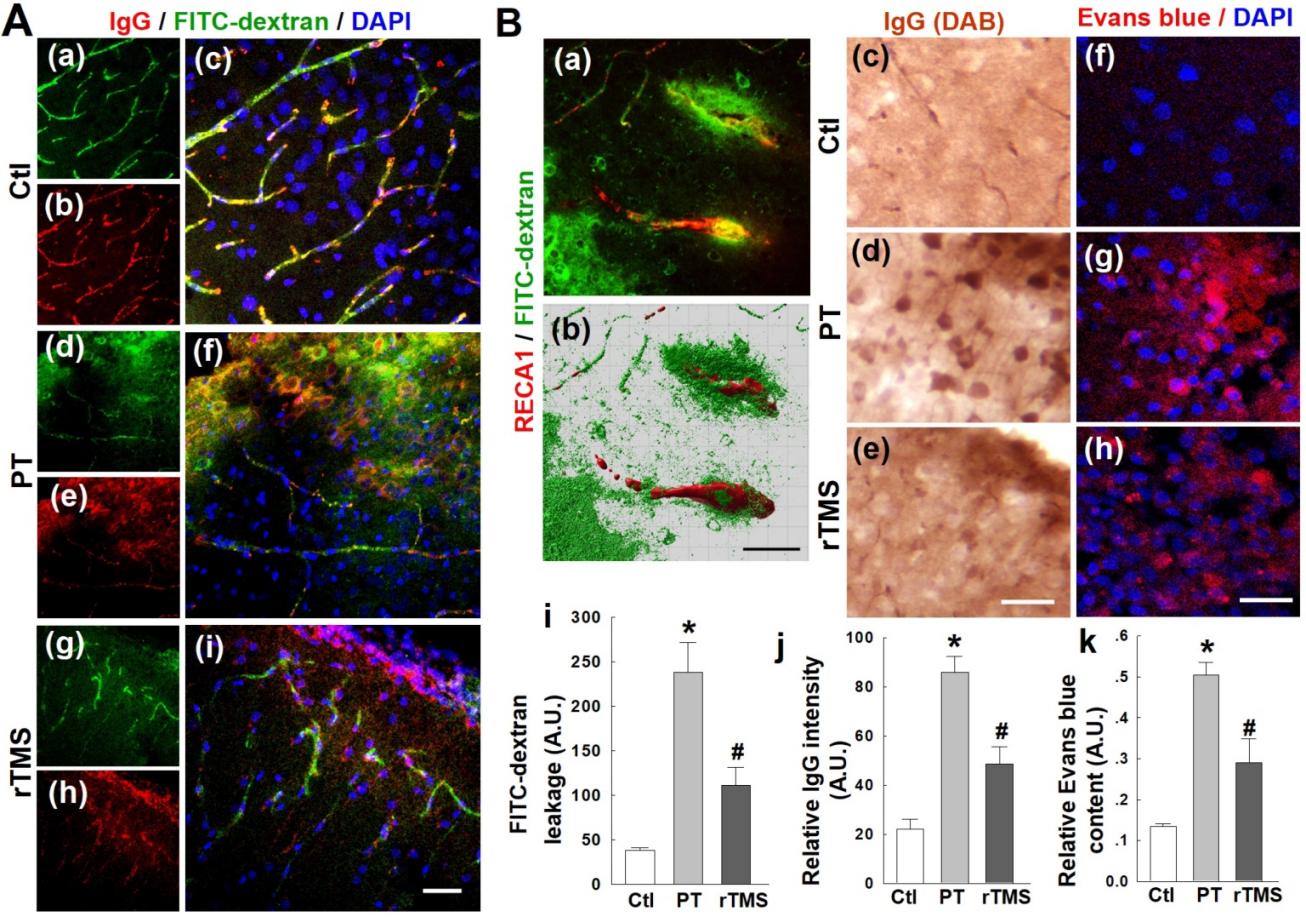
** rTMS treatment preserved blood-brain barrier (BBB) permeability induced by PT-stroke in the peri-infarct cortical region 6 days after PT stroke.** (A) Rat brains without saline flush. Representative confocal microscopy images of IgG staining (red in a-i) and FITC-dextran signal (green) in the peri-infarct areas. Nuclei were counterstained with DAPI (blue). (B) FITC-dextran and Evans blue injected rats were transcardially flushed with ice-cold saline, and the brain sections were subjected to confocal imaging or DAB-stained image acquisition. Representative 3-D reconstruction (Bitplane Imaris software) of typical FITC-dextran leakage in PT animals (green in a,b), IgG leakage (brown in c-e) and Evans blue (red in f-h) are shown. Quantitative analyses of extravascular FITC fluorescence (i) and IgG intensity (j) were performed. Evans blue penetration into brain tissue following intravenous administration was also quantified using peri-infarct protein samples (k) as indicated in *Methods*. Magnification: 40×, scale bar: 50 µm. *P < 0.05 versus Ctl group, ^#^*P* <0.05 versus PT group without rTMS treatment. Data are presented as means±SE (N = 5-8). A.U.: arbitrary unit.

Stroke, in humans or experimental animal models, causes substantial disruption of the BBB and leakage of blood components into the surrounding parenchyma. In this study, as expected, PT stroke caused extensive local dysregulation of BBB integrity. Representative confocal microscopy images of IgG staining and FITC-dextran signal in the peri-infarct region are shown in **Figure 1A.** Considerable leakage of FITC-labelled dextran was present in the cortical peri-infarct area of PT animals, but was abated by over 50% in rTMS group rats (**Figure 1B**). As depicted in **Figure 1B(c-e)** with representative confocal microscopy and DAB staining, PT stroke induced dramatic extravasation of IgG into the peri-infarct area, as quantified and shown in **Figure 1B(j)**. This was ameliorated substantially in rTMS-treated rats. Evans blue leakage was likewise increased in both PT and rTMS animals, but this leakage was greatly pronounced in PT animals that did not recieve rTMS, as shown in representative confocal images** (Figure 1B (f-h))** and data quantification using peri-infarct protein samples** (Figure 1B (k))**.

### Post-stroke expression of BBB tight junction components was protected by administration of rTMS

**Figure 2:**
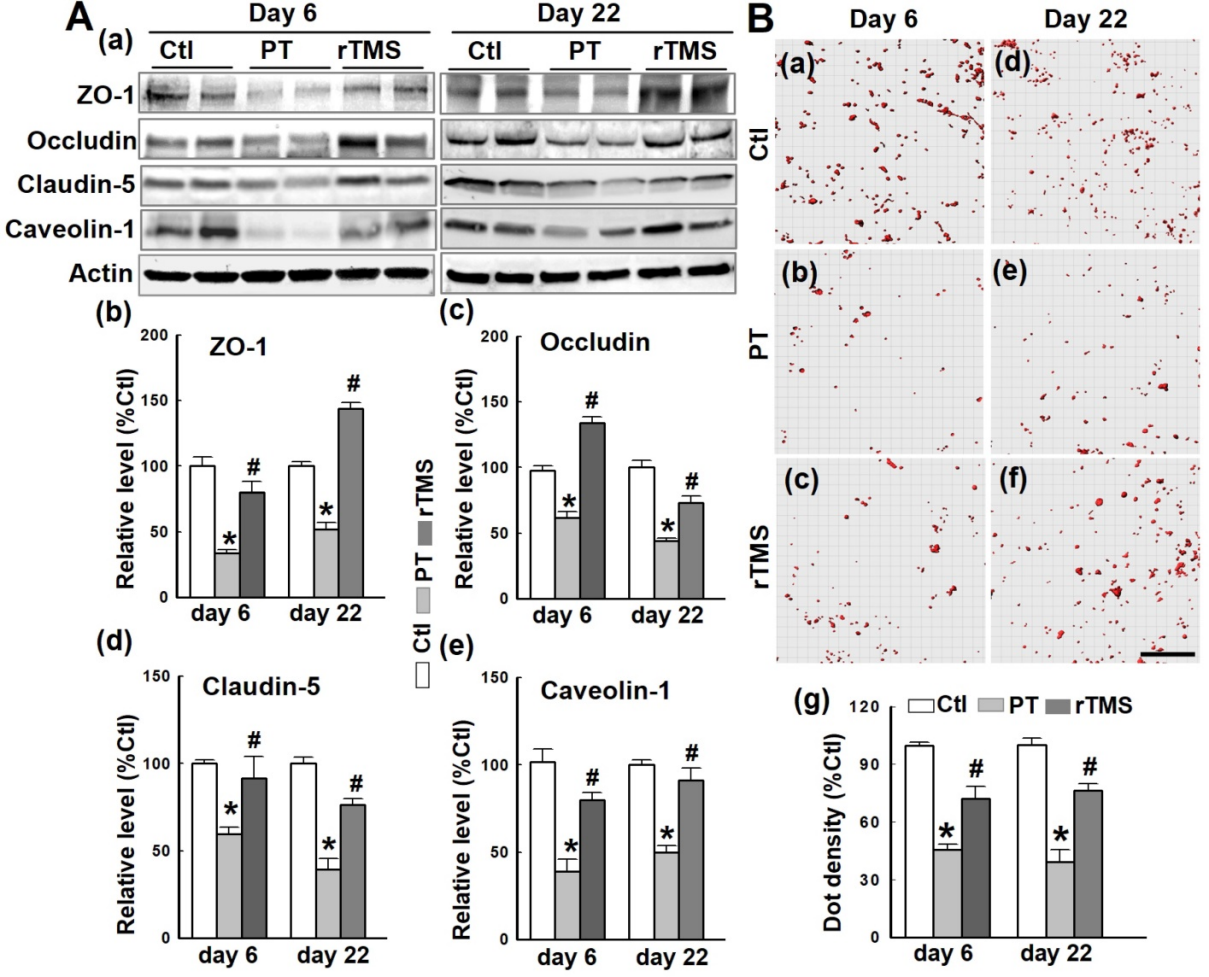
** rTMS treatment increased the expression of tight junction-associated proteins and scaffold protein, and promoted the interaction between ZO-1 and Claudin-5.** (A) Western blotting and quantitative analyses of ZO-1, occludin and claudin-5, and the scaffold protein caveolin-1 using control (Ctl) and peri-infarct cortical proteins at day 6 and 22 following PT stroke. (B) Duo-Link II *in situ* PLA immunostaining (a-f) and quantification (g) of the interactions between ZO-1 and claudin-5 in Ctl brain and peri-infarct areas at day 6 and 22. Red spots representing the bindings were surface separated and analyzed using Imaris software. Magnification: 40×, scale bar: 50 µm. Data are means±SE from 4-6 independent animals per group. *P < 0.05 versus Ctl group, ^#^*P* < 0.05 versus PT stroke group.

The BBB is composed of several components which, in concert, tightly regulate the efflux and influx of soluble compounds between systemic circulation and the brain. Western blotting and quantitative analyses demonstrated that the tight junction components ZO-1, occludin, claudin-5, and the endocytotic scaffolding protein caveolin-1 were all downregulated at both day 6 and day 22 after PT stroke **(Figure 2A)**. In contrast, rTMS caused upregulation of each of these components. The interaction of ZO-1 and claudin-5 was investigated using Duo-Link II *in situ* hybridization, as depicted in **Figure 2B.** The count of these interactions was reduced by PT stroke at both days 6 and 22, damage that was lessened significantly in rTMS group rats at both observed time points. These data suggest that rTMS post-treatment significantly preserved BBB tight junction assembly following PT stroke.

### rTMS treatment increased reoxygenation and perfusion of microvasculature and preserved vascular morphology in the peri-infarct region 22 days after PT stroke

**Figure 3:**
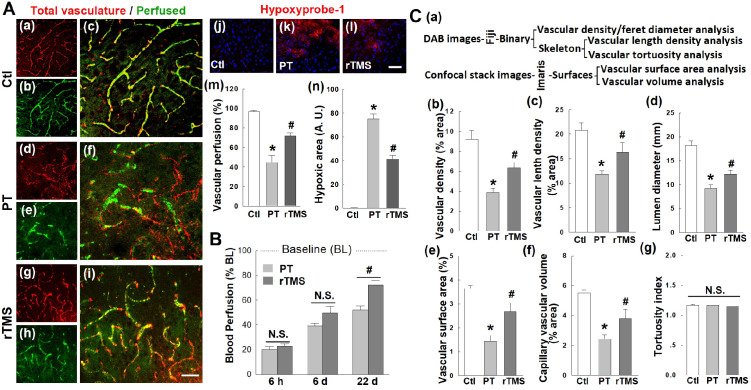
** rTMS treatment improved microvascular perfusion, local reoxygenation, and morphometric parameters in peri-infarct region examined 22 days after PT stroke.** (A) Labeling of RECA1 (a-i, red, represents total vasculature), FITC-dextran (green, represents perfused vasculature), and hypoxyprobe-1 probe (j-l, red, local oxygen gradient marker) in Ctl and peri-infarct regions. The percentage area of perfused vasculature compared to total vasculature (m), and hypoxic area (n) were quantitatively analyzed. (B) Regional blood flow at the surface of the infarcted area was also measured at three time points after stroke, using an Oxy-Lab Laser Doppler (see *Methods*). (C) Morphometric determination of the indicated vascular parameters in the peri-infarct zone 22 days after stroke. Schematic diagrams of techniques and software used for vessel quantification are shown in (a), and the comparative analyses are shown in (b-g). Magnification: 40×, scale bar: 50 µm. *P < 0.05 versus Ctl group, ^#^*P* < 0.05 versus PT group without rTMS treatment. Data are presented as mean ± SE from 5-8 animals in each group.

Vascular integrity in the peri-infarct region is compromised after human stroke, and this was mirrored in the PT stroke model. Vascular perfusion, a key measure of vascular function, was impaired in PT stroke animals compared to control group **(Figure 3A (a-i))**. Animals treated with rTMS had significantly greater perfusion at day 22, as measured via the percentage area of perfused vasculature (FITC-dextran staining) compared to total vasculature (endothelial marker RECA1) **(Figure 3A (a-i, and m))**. In line with these results, rTMS treatment restored tissue oxygenation. Using hypoxyprobe-1, a local oxygen gradient marker, PT stroke animals demonstrated dramatic tissue hypoxia in the peri-infarct region on day 22 **(Figure 3A (j-l))**. The total area of hypoxic tissue was nearly halved following rTMS treatment **(Figure 3A (j-l, and n))**. It should be noted that time-dependent restoration of vascular function and local reoxygenation was confirmed with laser dopplerimetry, which revealed no difference in cerebral blood flow between PT and rTMS animals at day 6 after PT stroke. A significant increase was observed, however, following rTMS treatment at day 22 after stroke (**Figure 3B**). Finally, vascular morphology was similarly impaired in animals receiving PT stroke in such metrics as vascular density, vascular branch density, lumen diameter, surface area, and capillary volume **(Figure 3C)**. These altered morphology metrics were significantly reversed by rTMS treatment. Tortuosity, a measure of twisting, was not affected by PT or rTMS at the examined time point.

### Vascular components and regulatory proteins were impacted by PT stroke but were upregulated by rTMS

**Figure 4:**
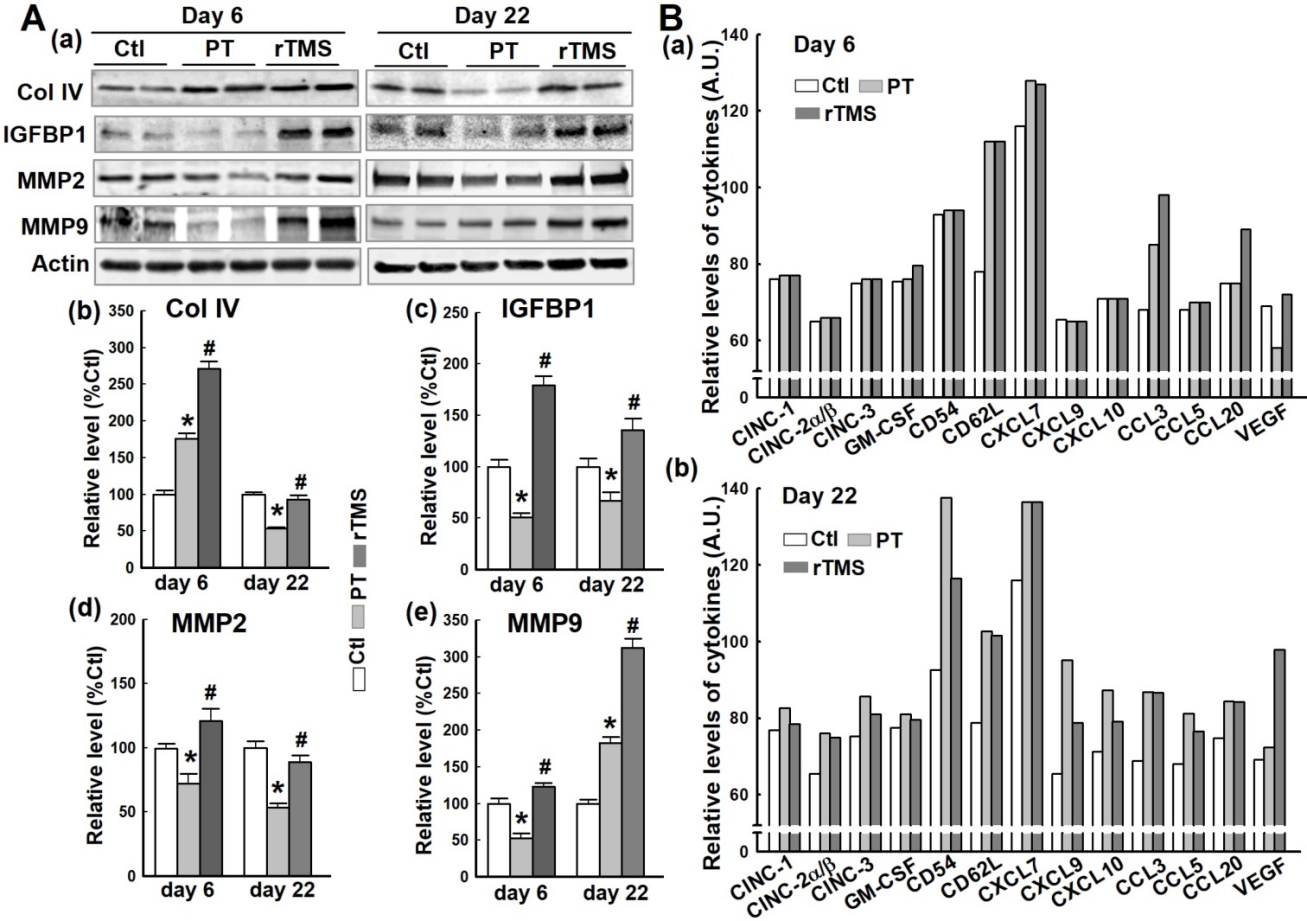
** Effects of rTMS treatment on the expression of vascular component and regulatory proteins, and cytokine levels at early and late stages following PT stroke.** (A) Western blotting and quantitative analyses of the indicated proteins associated with vascular structure components and the regulation of the endothelial permeability barrier were performed, using protein samples from the peri-infarct brain region at day 6 and 22 after PT stroke (a-e). (B) Expression of the indicated cytokines in Ctl and peri-infarct brain proteins, at day 6 and 22, was examined using Proteome Profiler Rat Cytokine Array Kits (see *Methods*). Data are means±SE (N = 4-6 animals/group). *P < 0.05 versus Ctl group, ^#^*P* < 0.05 versus PT stroke group.

Critical vascular components and regulatory compounds are damaged by stroke, contributing to BBB permeabilization and vascular failure. The protein collagen IV (Col IV), essential to vascular structure, was actually upregulated in the short-term after PT stroke **(Figure 4A (a, b))**. At day 22, however, this effect had reversed, leaving PT animals with significant attenuation of Col IV compared to Ctl. In contrast, Col IV was dramatically upregulated in rTMS group rats at day 6. On day 22, Col IV levels in rTMS group were greater than PT group and comparable with Ctl. Insulin-like growth factor binding protein 1 (IGFBP1) is associated with vascular repair, which was demonstrated to be impaired by PT stroke compared to Ctl animals at both the early and late phases **(Figure 4A (a, c))**. Animals treated with rTMS had markedly higher levels of IGFBP1 than both Ctl and PT at both time points. Matrix metalloproteinases are a pivotal part of the angiogenic response, degrading the extracellular matrix. PT stroke caused a significant downregulation of both MMP2 and MMP9 at day 6 and MMP2 at day 22, whereas rTMS animals displayed a stark increase in the levels of these proteases **(Figure 4A (a, d and e))**. At day 22, however, MMP9 levels broke with this trend, presenting a sharp increase in MMP9 levels in PT compared to Ctl. Rats in rTMS group demonstrated an even more drastic increase. Finally, using a Proteome Profiler Rat Cytokine Array Kit, cytokine levels were measured at day 6 and day 22 **(Figure 4B)**. At day 6, we found the levels of CD62L and CXCL7 were equally elevated in PT and rTMS groups, while stroke-induced CCL3 expression was further promoted by rTMS. CCL20 levels were not changed in Ctl and PT groups, but were increased by rTMS treatment. VEGF expression was decreased by PT stroke, and this effect was reversed by rTMS. At day 22, most of the measured cytokines were slightly (CINC-1, CINC-2α/β, CINC-3, GM-CSF and VEGF) or markedly (CD54, CD62L, CXCL7, CXCL9, CXCL10, CCL3, CCL5, CCL20) induced by stroke compared to Ctl. Of note, ischemia-induced elevation of CD54, CXCL9, CXCL10, and CCL5 levels could be apparently attenuated by rTMS **(Figure 4B (b))**. Intriguingly, VEGF expression at day 22 was further elevated following rTMS treatment.

### Post-stroke angiogenesis was promoted and sustained long-term by rTMS

**Figure 5:**
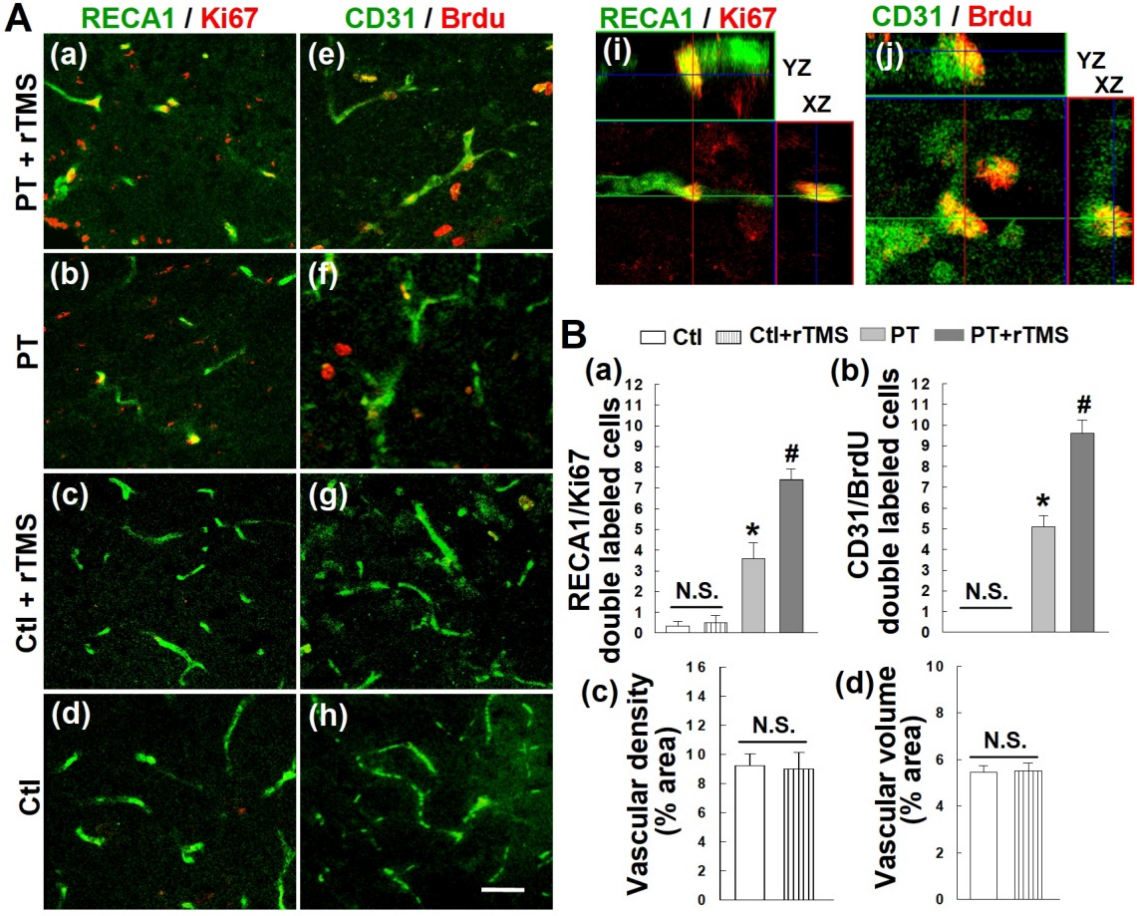
** rTMS treatment promoted post-ischemic angiogenesis, as observed on day 22 following PT stroke.** (A) Representative confocal microscopy images of endothelial cell antigen markers RECA-1 (a-d, green) and CD31 (e-h, green), as well as proliferative cell markers Ki-67 (a-d, red) and Brdu (e-h, red) in Ctl and peri-infarct areas 22 days after PT stroke. Orthogonal images are represented in the x-z and y-z directions (i, j). (B) Counting studies of double positive cells of RECA1/Ki67 and BrdU/CD31 colabeling are shown in (a, b). Vascular density and volume between non-stroke Ctl and rTMS treated Ctl group showed no significant (N.S.) differences (c, d). Magnification: 40×, scale bar: 50 μm. Data represent mean±SE (N=5-8). *P < 0.05 versus Ctl group, ^#^*P* < 0.05 versus PT stroke group without rTMS treatment.

Angiogenesis occurs as a response to both experimental and human stroke, but these newly-formed vascular beds are not long-lived and typically die off later in recovery. To first test whether rTMS could boost this angiogenic response, we stained for the vascular endothelial marker RECA1 and the cellular proliferation marker Ki67 **(Figure 5A (a-d))**. Colocalization of these two signals was interpreted as new vascular endothelial cells **(Figure 5A (i))**. This effect was further confirmed by injecting the synthetic thymidine analog BrdU, which incorporates into the DNA of newborn cells. Co-labeling for CD31, a vascular endothelial marker, and BrdU revealed a substantial increase in newly formed endothelial cells **(Figure 5A (e-h, j))**. The angiogenic response of these cells was significantly greater in rTMS group than PT stroke group **(Figure 5B (a, b))**. Further data demonstrated that there are no significant differences in angiogenic activity, as well as in vascular density and volume, between healthy, non-stroke rats and rTMS treated non-stroke group **(Figure 5A (c, d, and g, h), 5B (a-d))**.

**Figure 6:**
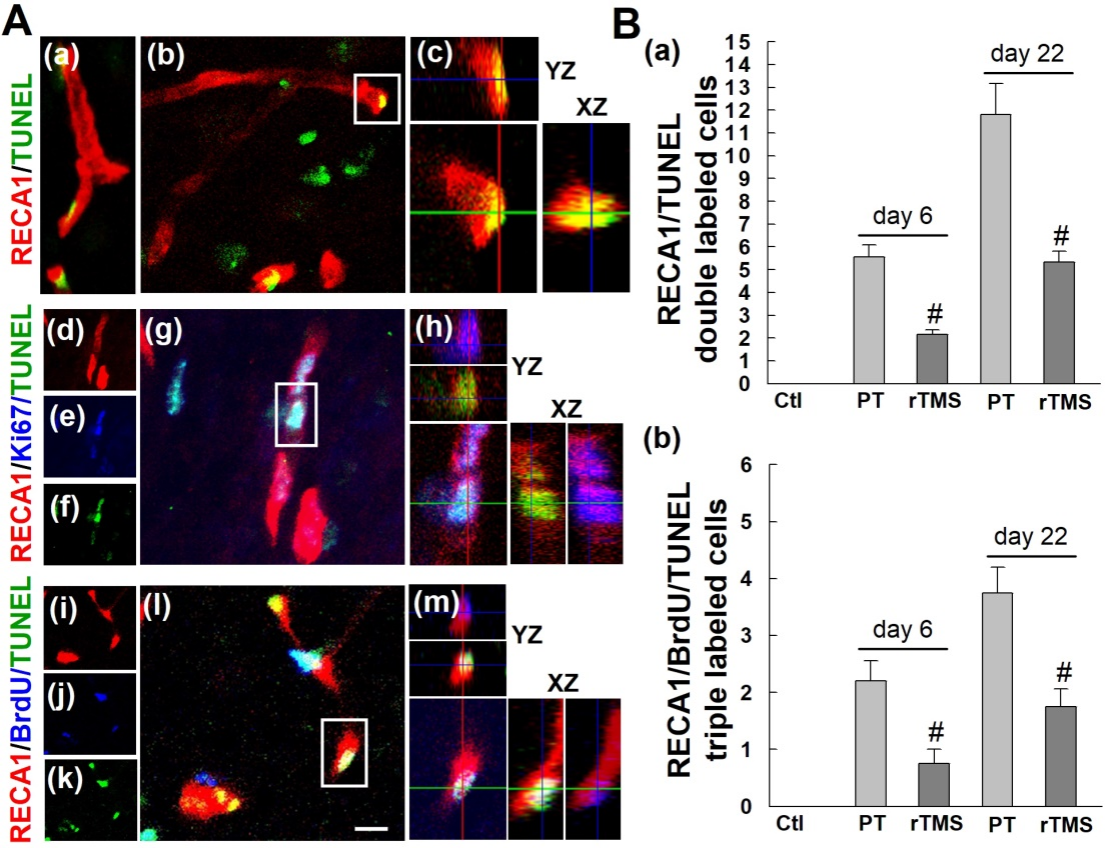
** rTMS treatment attenuated the apoptotic counts of existing and newborn endothelial cells in peri-infarct region examined at 6 and 22 days after PT stroke.** (A) Representative confocal images of the peri-infract cortex in the PT group stained with RECA1 antibody (a-m, red), Ki67 antibody (d-h), BrdU antibody (i-m, blue) and TUNEL (a-m, green). Confocal orthogonal view of zoomed (inset) image is shown to the right of each panel, indicating the co-localization of RECA1, RECA1/Ki67, or RECA1/BrdU with TUNEL labeled cells. (B) The numbers of apoptosis of total endothelial cells (RECA1/TUNEL double labeled cells) and the newborn endothelial cells (RECA1/BrdU/TUNEL triple labeled cells) were counted and statistically analyzed. White scale bar: 20 μm, magnification: 40×. Data are presented as mean ± SE from 5-8 rats in each group. ^#^*P* < 0.05 versus PT group without rTMS treatment.

To determine whether these new vascular beds were persistent over time, however, we examined apoptotic cell death of both new and existing vasculature at day 22 after stroke. The number of total apoptotic endothelial cells (RECA1/TUNEL double-labeled) were increased in PT group versus Ctl group at both the early and late phase after stroke induction, day 6 and day 22, respectively **(Figure 6A (a-c) and B(a))**. Application of rTMS was sufficient to reduce this cell death. Newly formed endothelial cells, as indicated by triple staining of RECA1/Ki67/TUNEL or RECA1/BrdU/TUNEL, followed the same trend **(Figure 6A (d-m) and B (b))**. PT stroke caused substantial cell death, but this was mitigated in rTMS group animals. Taken together, our data indicate that not only does rTMS promote the angiogenic response, but it can also sustain this response long-term.

### Excessive astrocyte-vasculature interactions after stroke were reduced by rTMS

**Figure 7:**
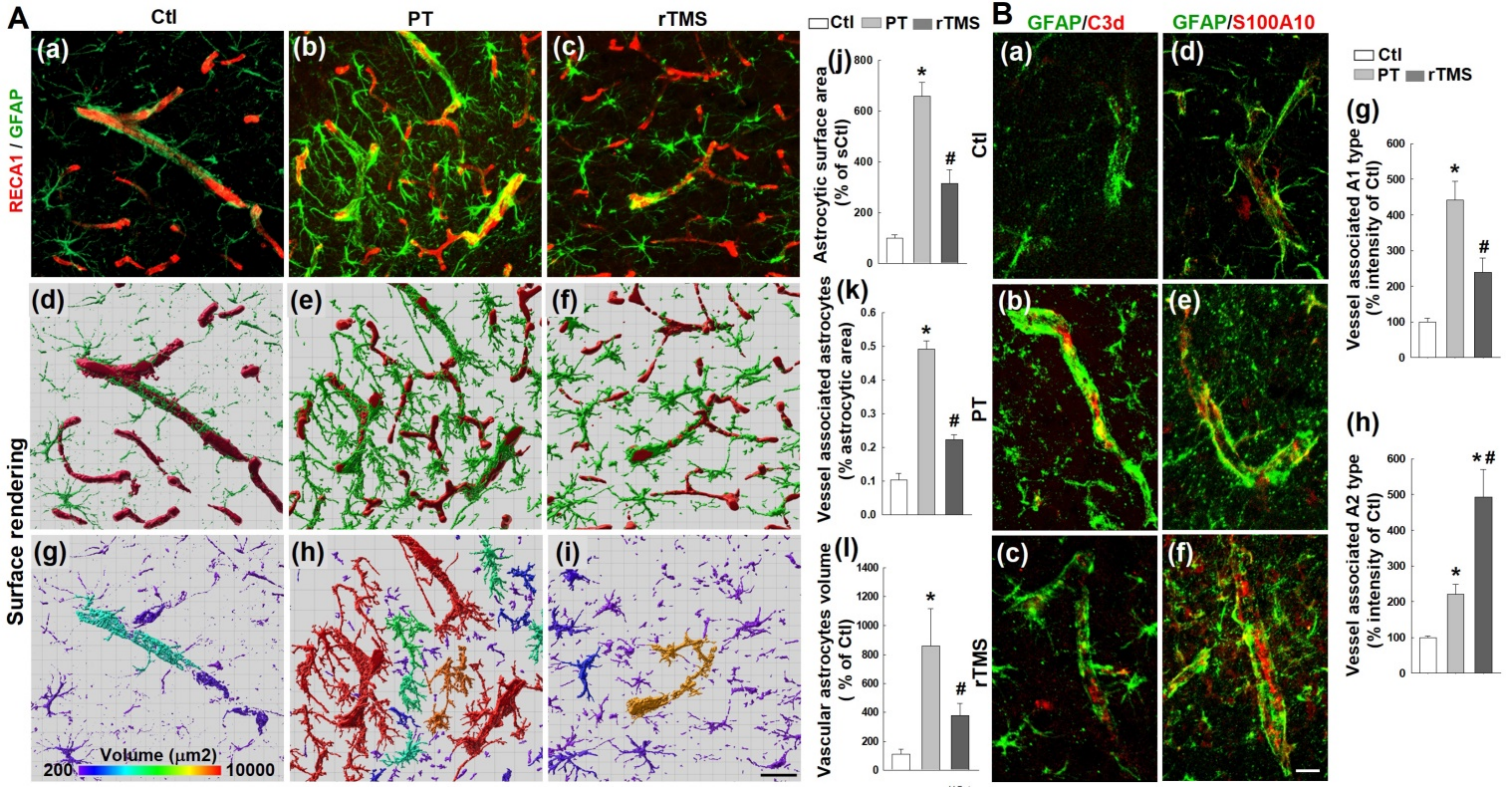
** rTMS treatment inhibited excessive astrocyte-vasculature interactions and induced vessel associated A1 to A2 switch of astrocytic phenotypes in the peri-infarct region examined at day 22 following PT stroke.** (A) Representative confocal microscopy images showing GFAP staining (a-c,green) and RECA1 staining (a-c,red) within the peri-infarct cortex on day 22 after stroke. Images were further 3-D processed (projection and surface rendering of GFAP and RECA1) and analyzed with Bitplane Imaris (d-l), and the total astrocytic surface area (j), vessel associated astrocytic area (k) and different morphological volumes of astrocytes (l) are shown, respectively. (B) Representative double immunofluorescence staining and immunoactivity analyses of vessel associated C3d (a marker of the toxic A1 phenotype, a-c and g, red) and S100A10 (a marker of the protective A2 phenotype, d-f and h, red) in the peri-infarct brain regions 22 days after PT stroke. Magnification: 40×, scale bar: 50 μm. Data are presented as mean ± SE, n = 6-8 per group. *P < 0.05 versus Ctl, ^#^*P* < 0.05 versus PT control group without rTMS treatment.

Astrocytes are activated in response to stroke and play a part in the recovery process and glial scarring. Peri-infarct regions were co-labeled with RECA1 and GFAP, confocal images were 3D reconstructed followed by surface rendering, areal and volumetric analysis using Imaris software. As revealed in **Figure 7A**, PT stroke induced a substantial increase in the total surface area of astrocytes, consistent with enlargement in activated astrocytes **(Figure 7A (a-j))**. As well, PT stroke animals had increased contact surface area and volume of astrocytes associated with vasculature **(Figure 7A (a-i, and k-l))**. This enlargement of both total astrocytes and those associated with cerebral microvessels was substantially attenuated in rTMS group animals, indicating a reduction in total activation that could lead to the attenuation of hampered vascular repair caused by glial scarring and spatial infiltration.

### Vessel-associated astrocyte polarization was shifted towards the A2 phenotype in rTMS treated animals

Activated astrocytes differentiate into one of two polarized phenotypes, the pro-inflammatory A1 state and the anti-inflammatory A2 state. The expression of vessel-associated C3d (a marker of the toxic A1 phenotype, **Figure 7B (a-c)** red) and S100A10 (a marker of the protective A2 phenotype, **Figure 7B (d-f)** red) were co-labeled with GFAP in the peri-infarct brain regions 22 days after PT stroke. Immunoactivity analyses demonstrated a marked shift towards the A1 phenotype in PT animals which was lessened in rTMS group rats **(Figure 7B (a-c, and g))**. In contrast, rTMS rats displayed a commensurate increase in A2 astrocytes compared to PT group animals **(Figure 7B (d-f, and h))**. This demonstrates that rTMS may shift the balance of microvessel-associated astrocytic polarization towards the pro-repair A2 phenotype which may contribute to the improved vascular outcomes in these animals.

### PDGFRβ expression was promoted by rTMS treatment in the peri-infarct A2-type astrocytes and associated with microvasculature examined at day 22 after PT stroke

**Figure 8:**
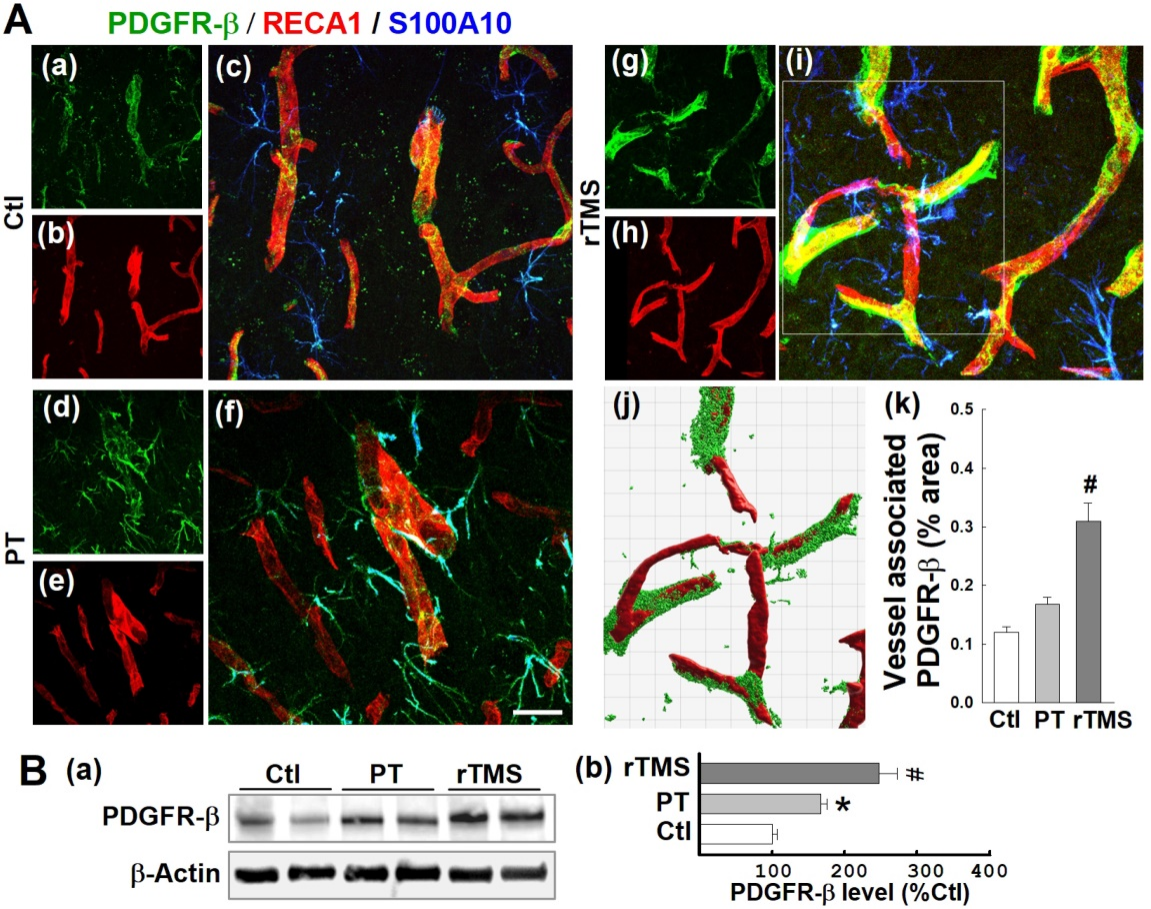
** rTMS treatment elevated PDGFRb expression associated with microvasculature and in the A2-type astrocytes in peri-infarct region examined at day 22 after PT stroke.** (A) Typical confocal microscopy images of PDGFRb (green), RECA1 (red) and S100A10 (blue, A2 phenotype marker) staining in the peri-infarct area 22 days following PT stroke (a-i). Using Imaris software, rTMS-treated group in the boxed region (i) was subjected to 3-D projection and surface rendering (j, green: PDGFRb, red: RECA1), and the vessel associated PDGFRb levels were analyzed and compared (k). (B) Representative Western blots (a) and quantitative analyses (b) of PDGFRb expression using protein samples from the peri-infarct brain region at day 22 after PT stroke. Magnification: 40×, scale bar: 50 μm. Data are presented as mean ± SE from 5-8 animals in each group. *P < 0.05 versus Ctl, ^#^*P* < 0.05 versus PT control group without rTMS treatment.

PDGFRβ is a receptor associated with vascular repair signaling. As demonstrated in representative confocal microscopy images taken at day 22, both vessel-associated (co-localized with RECA1) and A2-type astrocytic (co-localized with S100A10) PDGFRβ expression were slightly upregulated by stroke, an effect substantially amplified in rTMS treated animals** (Figure 8A (a-i))**. Vessel-associated PDGFRβ levels were analyzed and compared using 3-D projection and surface rendering using Imaris software. Quantitative results revealed no significant increases between PT and Ctl groups, but confirmed a significant increase of vessel-associated PDGFRβ after rTMS treatment** (Figure 8A (j, k))**. Western blot analysis, as depicted in **Figure 8B**, reiterated this pattern in total protein samples, wherein PT and rTMS groups exhibited elevated PDGFRβ levels compared to Ctl, and a significantly greater degree of amplification in rTMS-treated animals.

### rTMS treatment upregulated VEGF and TGFβ expression in vessel-associated A2-type astrocytes

**Figure 9:**
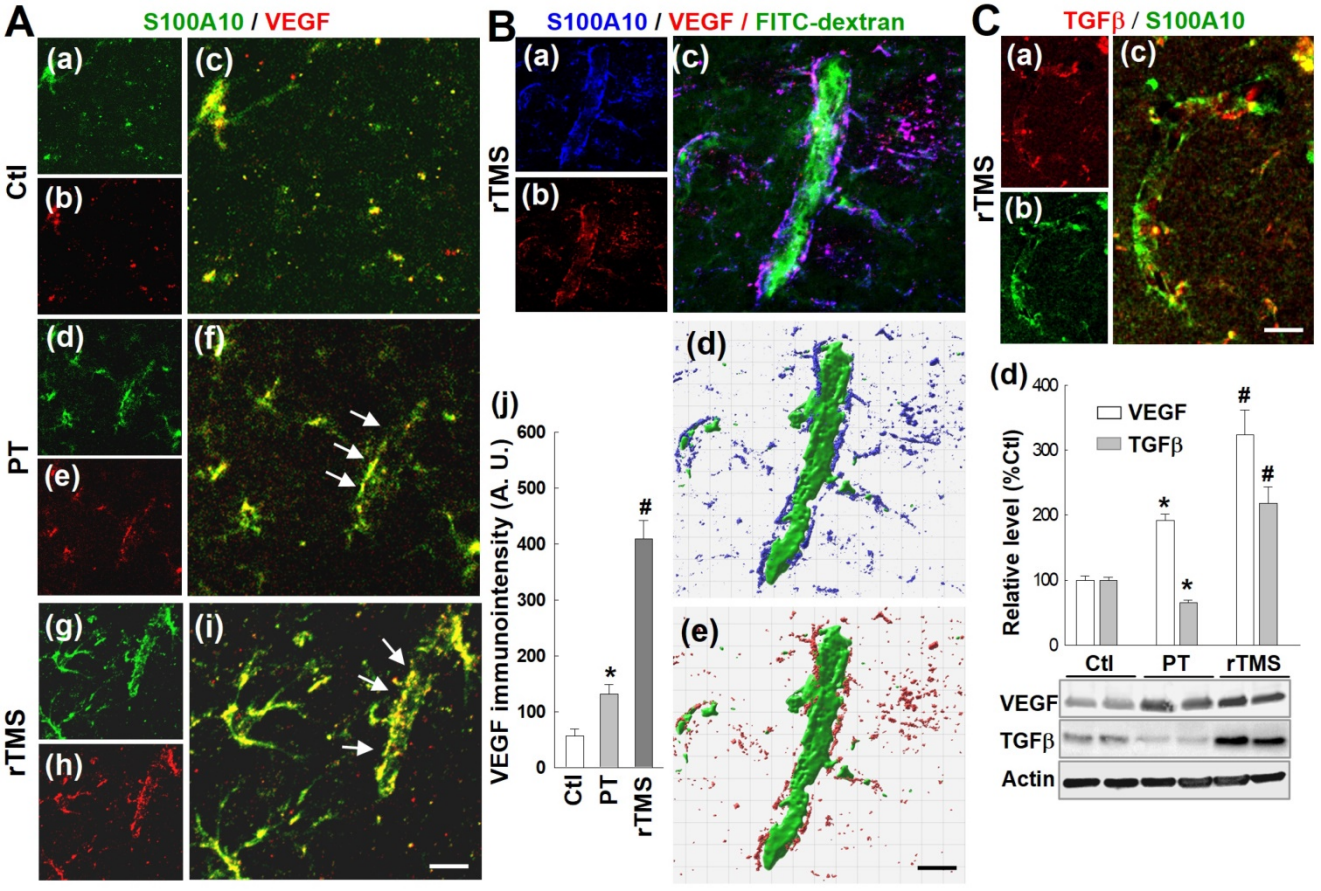
** rTMS treatment promoted post-ischemic vasculature expression of VEGF and TGFβ in A2-type astrocytes and the association with functional vasculature.** (A) Confocal images of S100A10 (green, A2 phenotype marker) and VEGF (red) revealing vascular expression (f, i, white arrows) of VEGF in A2 type astrocytes (merged yellow color) in the peri-infarct area 22 days after stroke. Quantitative analysis of VEGF immunointensity in Ctl, PT and rTMS groups (a-i) was performed and are shown in (j). (B) Representative confocal images showing FITC-dextran perfused blood vessels surrounded by A2 astrocytic VEGF distribution (a-c, note merged purple color) in the rTMS group. Imaris-aided 3D reconstruction showing tight association of A2-type astrocytes and VEGF distribution along with functional micro-vessels (d,e). (C) Representative confocal microscopy of vasculature labeled with TGFβ (a-c, red) and S100A10 (a-c, green) in the post-ischemic peri-infarct areas with rTMS treatment. Western blotting and data analyses of TGFβ and VEGF using peri-infarct protein samples 22 days after PT stroke (d). Magnification: 40×, scale bar: 50 μm. Data represent mean±SE (N = 5-8 in A & B, and N = 4-6 in C). *P < 0.05 versus Ctl. ^#^*P* <0.05 versus PT group without rTMS treatment.

Vascular endothelial growth factor, VEGF, is a key regulator of angiogenesis and plays a critical role in the vascular repair process after stroke. Representative images in **Figure 9A (a-i)** demonstrate that rTMS group rats have a markedly upregulated co-localization of A2-type astrocyte marker SA100A10 and VEGF on vascular structures, suggesting that A2-type astrocytes might release or cluster VEGF onto the vasculature. Immunointensity analysis, as depicted in **Figure 9A(j),** revealed that VEGF levels were dramatically increased in rTMS animals, compared to the relatively modest increases in PT animals. Representative images Imaris-aided 3D reconstruction illustrated the tight spatial coupling of A2 astrocytes and VEGF with FITC-dextran perfused functional vasculature **(Figure 9B)**. Additional representative confocal microscopy and Western blot analysis indicate that TGFβ, in contrast with VEGF, was attenuated in PT animals compared to both control and rTMS groups. rTMS animals however displayed dramatic elevations in both VEGF and TGFβ levels.

### Vascular expression of HIF-1α was dramatically upregulated in the peri-infarct region of rTMS-treated rats

**Figure 10:**
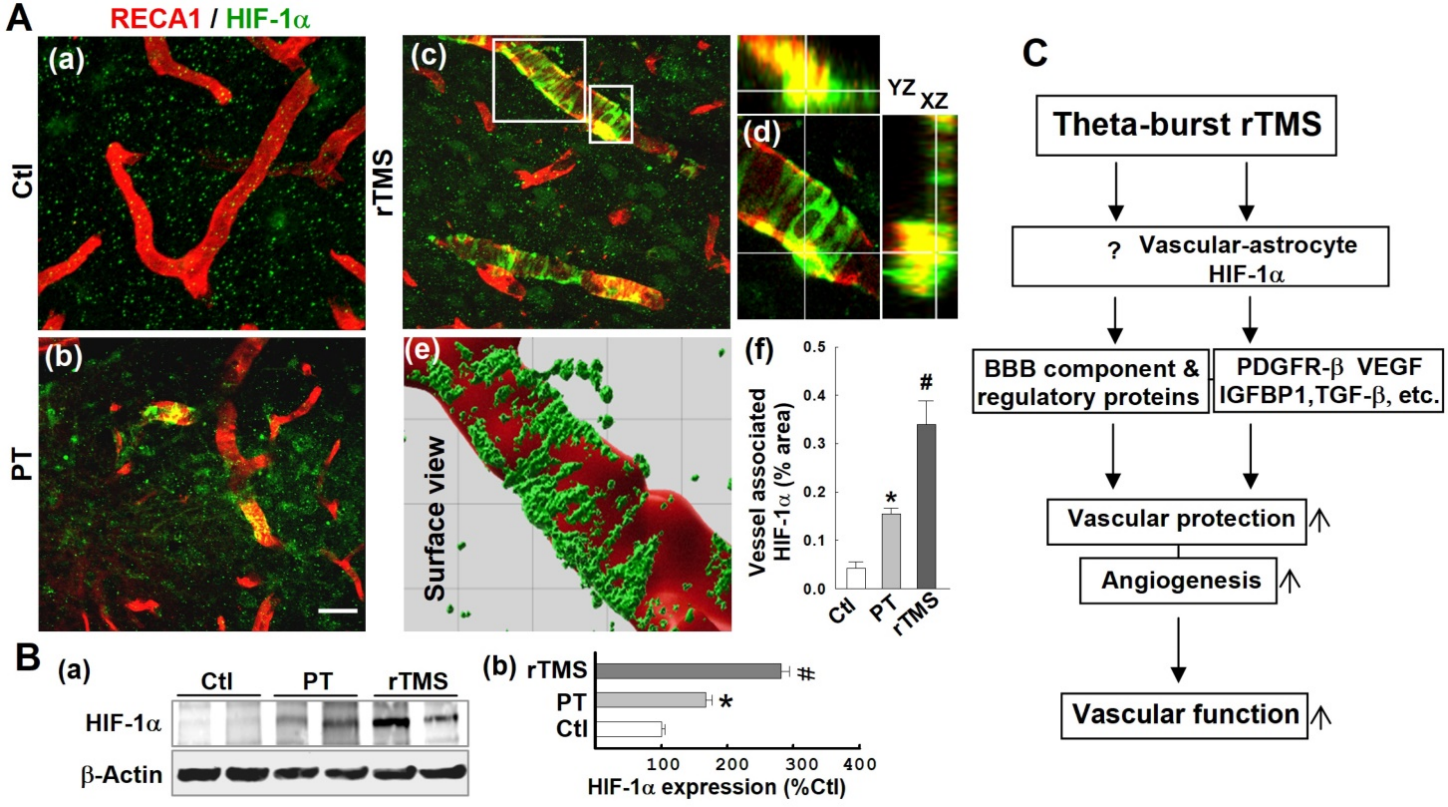
** rTMS treatment promoted vasculature expression of HIF-1α in the peri-infarct region examined at 22 days after PT stroke.** (A) Typical confocal microscopy images of RECA1 (a-d, red) and HIF-1α (a-d, green) staining in the peri-infarct cortical area showing HIF-1α expression profiles. Orthogonal view of zoomed (small inset in c) image showed in the right panel (d) revealing that HIF-1α expression is localized in the vasculature. rTMS-treated group in the boxed region (large inset in c) was further processed using Imaris software, and HIF-1α spatial relationship with vasculature are shown in (e). The vessel associated HIF-1α levels from each group were analyzed and compared with the aid of Imaris software (f). (B) Western blotting (a) and quantitative analyses (b) of HIF-1α expression in the peri-infarct protein samples 22 days after stroke. Magnification: 40×, scale bar: 50 μm. Data are mean ± SE (N = 5-8 in A and N = 4-6 in B). *P < 0.05 versus Ctl, ^#^*P* < 0.05 versus PT control group without rTMS treatment. (C) Schematic summary of the beneficial effects of rTMS therapy and the postulated underlying mechanisms of rTMS treatment in improving vascular protection, angiogenesis and finally, vascular function (see manuscript for detailed description).

HIF-1α is the master regulator of the hypoxic response and is reported to be upstream of PDGFRβ, TGFβ, and VEGF. To investigate potential mechanisms by which the various pathways affected by rTMS were regulated, we investigated the expression patterns of HIF-1α in each group of animals. As shown in **Figure 10A (a-f),** confocal microscopy and Imaris-aided analyses revealed that both PT and rTMS group rats displayed significantly upregulated vessel-associated HIF-1α expression compared to control group, while rTMS induced a sharply greater increase when compared to PT group. This was further confirmed with Western blot analysis, which reiterated previous findings that HIF-1α is increased in both PT and rTMS groups, to a greater degree in rTMS-treated animals **(Figure 10B)**. The beneficial effects of rTMS therapy and the potential underlying mechanisms of theta-burst rTMS treatment in improving vascular function are summarized in the schematic diagram **Figure 10C.**

### rTMS treatment preserved neuronal morphology and synaptic structure and protected behavioral outcomes

**Figure 11:**
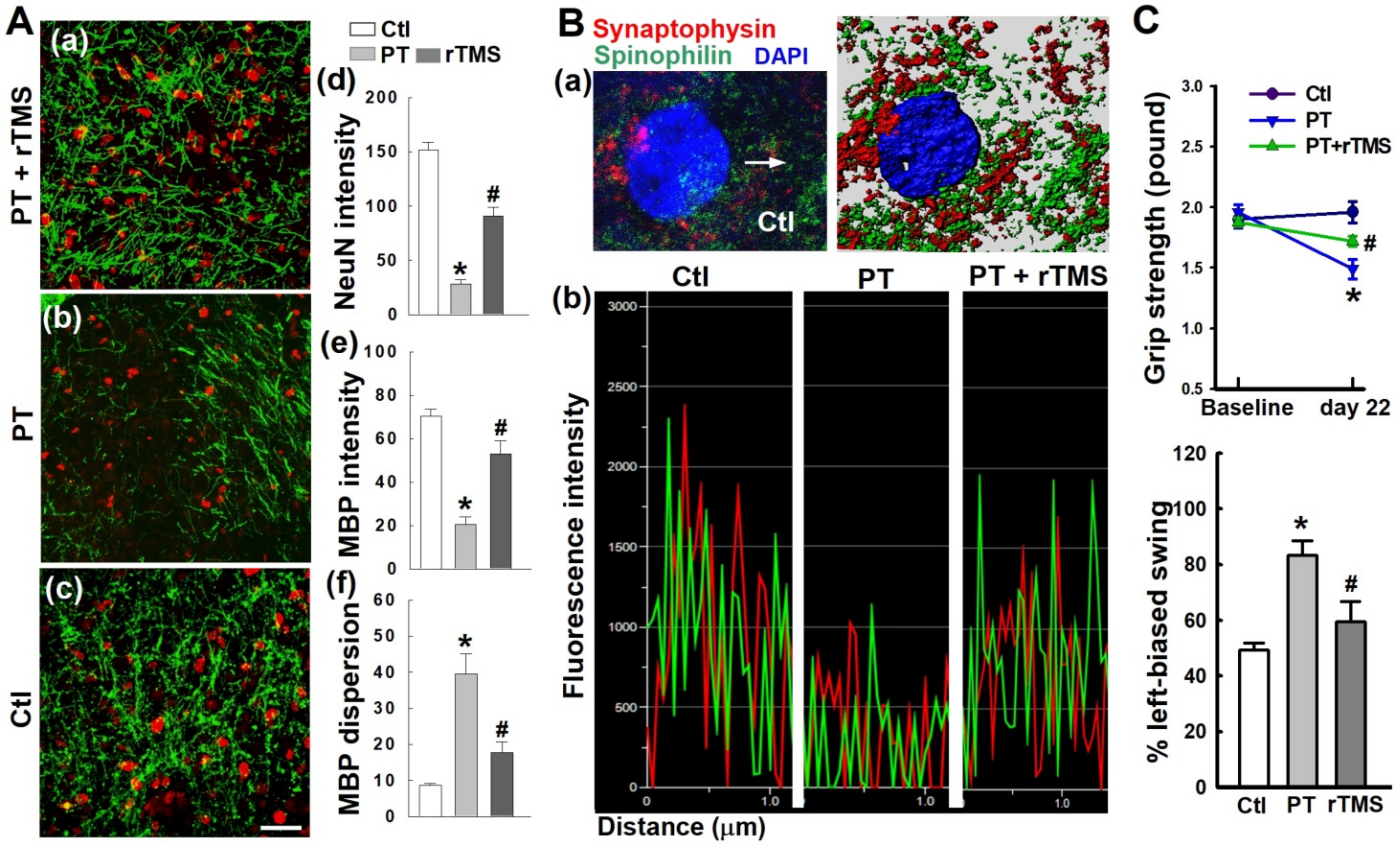
** rTMS treatment preserved neuronal morphology, synaptic structure integrity, and behavioral outcome 22 days after PT stroke.** (A) Immunofluorescence staining of NeuN and myelin basic protein (MBP) was performed. Histological analysis showed rTMS increased staining for NeuN and MBP and decreased MBP dispersion in the peri-infarct area. (B) Representative fluorescence co-staining of the markers of presynaptic synaptophysin (red), dendritic spinophilin (green), and DAPI (blue), and a typical Imaris-rendered image in the peri-infarct area 22 days after PT stroke (a). Graphs represent fluorescence profiles displaying immunointensities and co-localization between the two stained pre/postsynaptic granules (b). (C) Functional performance of rats was measured by grip strength test and left-biased swing test 22 days after PT stroke (d). Magnification: 40×, scale bar: 50 μm. Data represent mean ± SE (N = 4-6 in A & B, and N = 6-8 in C). *P < 0.05 versus Ctl. *^#^P* <0.05 versus PT group without rTMS treatment.

PT stroke resulted in significant attenuation of neuronal intensity, MBP network dispersion, and myelin disassembly in the peri-infarct cortical region, as illustrated in** Figure 11A**. However, these detrimental effects were effectively inhibited following rTMS treatment. Further study demonstrated that rTMS remarkably reduced damage to synaptic structure in the peri-infarct region, as evidenced by analysis of the fluorescence intensity profiles of spinophilin (a dendritic spine marker), and synaptophysin (a presynaptic marker), shown in **Figure 11B**. The data suggest that synaptic plasticity was preserved following rTMS application, a phenomenon which could be closely linked with functional improvements after stroke. Indeed, behavioral studies demonstrated that rTMS improved functional outcome as measured by behavioral tasks, including the grip strength test and the left-biased swing test **(Figure 11C).** Our data clearly suggests that rTMS treatment positively influenced neuronal function in the peri-infarct region which is closely correlated with a robust improvement in somatosensory and motor performance after PT stroke.

## Discussion

In this study, we demonstrated that rTMS applied after an experimental model of stroke can impart a wide array of protective effects on the cerebral vasculature which act synergistically to promote profound vascular recovery and regeneration. Using the photothrombotic stroke model, a widely used experimental animal stroke model that uses a photoactive dye and light to generate a targeted ischemic surrounded by a thin layer of salvageable penumbral tissue, we found that rTMS treatment protected against profound damage to the BBB and its components 50. Peri-infarct microvasculature, damaged by PT stroke, was morphologically protected and functional in rTMS group animals, increasing local tissue oxygenation. Vascular components and factors related to vascular repair were likewise elevated by rTMS. In response to PT, a complex and time-dependent cytokine response took place, with maximal differences observed 22 days after stroke. rTMS selectively ameliorated increases in cytokines promoting infiltration of peripheral immune cells while promoting those which possibly mobilize the migration and proliferation of epithelial progenitor cells. In addition, we found that rTMS induced a dramatic increase in vessel-associated A2 astrocyte polarization, along with a decrease in A1 astroglial expansion. rTMS treatment further promote vasculature-associated expression of TGFβ, VEGF, and PDGFRβ alongside sustained functional angiogenesis in the peri-infarct area. Finally, mechanistic work revealed that the diverse effects of rTMS were all accompanied with robust promotion of HIF-1α signaling by rTMS.

We first examined the effects of rTMS on the BBB. The BBB is an active barrier that tightly regulates transit between circulation and the CNS, and is dysregulated during stroke. Opening of the BBB is beneficial to some degree, as it allows peripheral immune cells to infiltrate and clean up necrotic debris, but prolonged BBB permeabilization is associated with cerebral edema, an excessive and prolonged neuroinflammatory response, and poor behavioral outcomes 13, 14, 53. Several experimental therapeutics have reduced post-stroke permeabilization of the BBB and resultant neurotoxicity, including carbon nanomaterials, antibiotics, immunoglobulin, and gonadal hormones 54-58. Likewise, our current results demonstrate that rTMS prevented the loss of BBB integrity after PT stroke. Rats treated with rTMS had substantially reduced BBB permeability causing reduced extravasation of dextrans and small molecules. The leakage of dextrans into brain tissue is associated with the degradation of critical BBB components, ZO-1, Occludin, and claudin-5, all of which were downregulated in both the early and late phase after PT stroke 53, 59-61. Levels of these components were preserved, however, in animals treated with rTMS. Upregulated vascular PDGFRβ expression, which is known to be associated with BBB integrity by way of preserving tight junction protein components, was observed in rTMS rats 62. Our previous work demonstrating that rTMS decreased inflammation and reactive oxygen species production, known contributors to tight junction damage, may also be an important driving factor behind its protective effects on the BBB 41, 63.

Stroke is a profound vascular injury, representing a catastrophic failure of the cerebral vasculature. Therefore, preservation and repair of the vasculature is imperative for recovery. We found that rTMS had multifactorial protective effects on the cerebral vasculature. Microvascular structure was particularly impacted by PT stroke, but animals treated with rTMS displayed improved vessel morphology and oxygen delivery. This is critical, as improved oxygenation is imperative to penumbral repair 64, 65. On the molecular level, the critical basement membrane component collagen IV was promoted in the short-term and preserved in the long-term by rTMS treatment 66. Further investigation revealed that rTMS activated multiple endogenous vascular repair mechanisms. Insulin-like growth factor binding protein 1 (IGFBP1) is a circulating factor that is integral in regulating insulin-like growth factor levels and is related to vascular outcomes in diabetes, which is known to impair neurovascular morphology 18, 67. In addition, IGFBP1 also triggers vascular repair via stimulating endothelial cell proliferation, growth, and expression of integrins 18. rTMS likewise boosted IGFBP1 in total protein samples. Upregulation of IGFBP1 therefore likely played an angiogenic role as well as a reparative one, an observation supported by previous reports that the increased levels of the protein increased neovascularization in a glioblastoma model 68, 69. Another pathway to vascular repair relies upon the actions of PDGFRβ. Transplantation of PDGFRβ overexpressing endothelial progenitor cells into mice with vascular injury caused dramatic revascularization and mitigated apoptotic cell death 19. In the middle cerebral artery occlusion (MCAO) mouse model, PDGFRβ KO mice had increased infarction volume, BBB damage, and pericyte cell death 70. Curiously, the loss of PDGFRβ was not associated with changes in angiogenesis 70. In the current study, PT induced a minor increase in PDGFRβ levels, but only rTMS animals displayed a significant increase in vessel-bound PDGFRβ. This bears out with our data regarding BBB integrity, vascular structure, and apoptotic cell death, as well as the improved neurological outcomes observed in our previous work 41. In addition, PDGFRβ overexpression is well-known to promote the process of angiogenesis 20.

Angiogenesis is a process by which new vessels are generated from existing vasculature in response to signals released by nearby hypoxic tissues, namely certain cytokines and trophic factors like VEGF 71, 72. These proteins act to stimulate proliferation and migration of local and peripheral endothelial progenitor cells. VEGF, in concert with angiopoietin, is upregulated in regions of new, functional vasculature after stroke 72, 73. Our results indicated that VEGF levels were upregulated in rTMS animals, which coincided with the presence of new blood vessels. For the process of angiogenesis to take place, the surrounding extracellular matrix must be degraded to facilitate migration and to carve a path for the nascent vessel to follow. Members of the matrix metalloproteinase family (MMP), particularly MMP2 and MMP9, are secreted by infiltrating leukocytes for this very purpose 73, 74. Our work demonstrates rTMS is associated with persistent elevation of MMP after PT stroke. While excessive levels of MMP production are associated with BBB permeabilization and inhibition of angiogenesis, the presence of long-term angiogenesis and the absence of exacerbated BBB extravasation informs us that this activation did not reach this dangerous threshold 74, 75. An important point to note, however, is that it is not entirely clear whether the neovascularization present is angiogenesis or vasculogenesis, a process that generates new vasculature and is predominate during embryonic development 76. While most post-stroke neovascularization is indeed angiogenesis, evidence suggests that vasculogenesis can occur after hypoxic brain injury under certain conditions 76, 77. Further work should be done to delineate the origins of the new vasculature formed after stroke and to what degree each pathway is affected by rTMS. Regardless of their origins, it is debated whether these new blood vessels truly persist long-term, as it is argued that new vascular beds generated after stroke primarily serve to aid in the clean-up process by facilitating neutrophil infiltration 24. In this current study however, endothelial apoptosis of both existing and new cells is reduced both in the short-and long-term after stroke rTMS animals compared with those in PT group. Thus, these results demonstrate that not only is rTMS vasculoprotective for existing vessels, but that it can protect the new and vulnerable vasculature generated in the peri-infarct region after stroke. As demonstrated in our previous work, this protection is likely due, in part, to the anti-inflammatory and anti-oxidative effects of rTMS on the neuronal microenvironment 41.

Cytokines and chemokines are key mediators of inflammation, inducing complex cellular responses and coordinating the action of immune cells to protect against infection and mobilize cellular recovery efforts 78. Stroke ignites a complex inflammatory response; one that our previous work demonstrated is abated by rTMS 41. In the current work, a separate set of cytokines was investigated, and several important trends emerged over the course of the post-stroke recovery period. In the early phase, 6 days after stroke, there were relatively few changes in the cytokines studied. CD62L and CXCL7, which facilitate neutrophil extravasation and endothelial progenitor cell binding respectively 79, 80, were equally upregulated in rTMS and PT group animals. CCL3 and CCL20, which are associated with leukocyte infiltration and angiogenesis, were the only cytokines upregulated by rTMS beyond that of the PT group 81. More diverse and greater differences between groups, however, emerged in the late phase, 22 days after PT stroke. PT induced long-term elevation of many of the measured cytokines that are associated with endothelial inflammation and immune cell infiltration into the CNS. The downregulation of several of these (CXCL10, CD54, CXCL9, and CCL5) by rTMS treatment may be contributing to the overall improvement in both vascular and neurological outcomes by decreasing peripheral immune cell recruitment and infiltration to the peri-infarct cerebral vasculature 78, 81-84. This is in line with human results in the clinic. For example, the persistent increase of soluble CD54 has been found in stroke patients, suggesting leukocyte-mediated brain damage occurs in response to acute ischemic stroke 84. By abating this response, the cerebral microvasculature may be better equipped for self-preservation and repair.

One potential target that mediates the brain's immune response is the astrocyte. Astrocytes are one of the key components of the BBB and play a key role in regulation and maintenance of the BBB and neurovascular microenvironment 85. In response to ischemic injury, astrocytes are activated by reactive microglia and begin forming a glial scar to sequester the infarcted area and prevent toxic necrotic debris from further damaging the surrounding area 86. While there are indeed benefits to glial scarring, the process does not completely sequester necrotic liquefaction and can hamper recovery efforts when taken to excess 87, 88. That said, some degree of astroglial scarring can actually support regenerative efforts 89. It is then encouraging that rTMS reduced, but did not eliminate, the volume of vessel-associated peri-infarct astrocytes. Such a reduction, but not complete reversal, could permit and even encourage recovery in the area. Upregulation of TGFβ, associated with glial scarring, in both PT and rTMS group animals may relate to this phenomenon, but why increased levels of TGFβ are present in rTMS rats which display less glial expansion is unclear 90.

Astrocytic activation is not a unipolar phenomenon, however. Upon activation via signals from reactive microglia, astrocytes polarize into one of two phenotypes, the pro-inflammatory A1 phenotype and the anti-inflammatory A2 phenotype 91. The A1 phenotype, as noted above, serves to release inflammatory factors and upregulate genes that are associated with synaptic degradation. A2 phenotype astrocytes, however, upregulate anti-inflammatory cytokines and trophic factors, serving to enhance cell survival and synaptic integrity in their vicinity 91. Our work demonstrated a marked shift in polarization in vessel-associated astrocytes from the A1 to A2 phenotype. The association with this A1 to A2 shift and improved outcomes has been demonstrated in a study utilizing the MCAO mouse model and our previous work 41, 92. Intranasal delivery of Wnt-3a attenuated A1 astrocytic polarization while promoting A2 polarization, reducing infarction volume and peri-infarct apoptosis after MCAO induction 92. Intriguingly, Wnt-3a treated animals underwent a similar shift in microglial polarization as well, shifting from the pro-inflammatory M1 state to the anti-inflammatory M2 state 41, 92, 93, in agreement with findings in our previous work. Activated inflammatory microglia push astrocytic polarization to the A1 state preferentially, so the rTMS-induced shift in microglial polarization may be, in part, responsible for the preponderance of A2 astrocytes we observed in rTMS-group rats 41, 94. Further microscopy examining the aforementioned rise in VEGF found a dramatic rise in astrocyte-associated VEGF signal in rTMS animals. Astrocyte-derived VEGF is associated with increased tube formation and resistance to hypoxia in cell culture, although some animal studies have found it can impair BBB function 74, 95. It is not clear if the astrocyte-associated VEGF signal is indeed astrocyte-derived, but reduced impairment of the BBB in rTMS animals suggests that either it is derived from another cell type or that the other myriad effects of rTMS outweighed any potential BBB damage.

While all of the different pathways involved in rTMS-induced vascular recovery are indeed interconnected, we sought after a deeper underlying mechanism that could tie these findings together. Several of the effectors of vascular recovery and preservation of BBB components are downstream from the previously noted proteins TGFβ, VEGF, IGFBP1, and PDGFRβ 62, 90, 96-98. HIF-1α, the master regulator of the hypoxic response is positioned upstream of all four of these targets and was promoted by rTMS treatment after PT stroke. The TGFβ pathway, responsible for increased collagen deposition, is regulated and activated by HIF-1α 99. VEGF is highly induced by HIF-1α signaling, although the maladaptive effects of excessive astrocyte-derived VEGF are curiously not affected by HIF-1α knockdown 95, 100. This could partially explain why dramatic increases in vascular HIF-1α failed to cause any notable failure of BBB integrity. Finally, PDGFRβ signaling is known to be induced by HIF-1α via IL-6 activation 101. Thus, it appears that HIF-1α is a key driving force of the various mechanistic pathways activated by rTMS treatment, as depicted in Figure 10C. HIF-1α is known to be expressed in the peri-infarct region after stroke, although whether this is always beneficial or sometimes maladaptive is disputed 101 In this study, however, the pathways activated by HIF-1α clearly worked synergistically to promote powerful recovery mechanisms. Future work should focus on pinpointing the mechanisms by which HIF-1α is induced by rTMS and how potential detrimental effects of HIF-1α signaling are abated after rTMS treatment.

We also examined fine neuronal morphology and synaptic measures to determine how rTMS influences neuronal function at the behavioral level at the late stage after PT stroke. Our study indicates that rTMS-induced vascular protection and neovascularization is associated with the attenuation of neuronal and synaptic structure damage in the peri-infarct area. This is consistent with the classically accepted concept that stroke-induced functional deficits are closely related to impaired synaptic plasticity, as reflected by injured neurons and synaptic proteins. We have previously demonstrated that the lesion volume can be significantly reduced following rTMS treatment 3 weeks after stroke 102. The protective role against PT stroke of rTMS could be multi-factorial, wherein rTMS treatment can alter the pathophysiology of stroke itself by protecting neurons and attenuating lesion volume, promoting nerve repair and functional integration, protecting vasculature, and promoting neovascularization. Our current data suggest that improvement of vascular function by rTMS can positively influence neuronal function following stroke.

It is well established that GABAergic interneurons and glutamatergic cells have diverse actions following cerebral ischemia 103. GABA-mediated tonic inhibition promotes recovery in the acute phase after stroke, while it exacerbates brain injury during the recovery phase of stroke 104. In addition, GABA-mediated phasic inhibition has been demonstrated to play a beneficial effect both in acute and chronic phases of ischemic stroke 105, 106. In a PT stroke mouse model, theta-burst rTMS primarily activates GABAergic interneurons and elevates phasic inhibition without impacting tonic inhibition in the peri-infarct cortex 107. Therefore, the potential GABAergic effects on endothelial progenitor cells and angiogenesis in stroke recovery still need to be elucidated.

In the current work, rTMS was continuously delivered by targeting the infarcted hemisphere using a pair of Helmholtz coils. There are potential limitations of this treatment method for small animals in this study. Although the center of the magnetic field from the Helmholtz coils was focused to the whole injured hemisphere, we could not rule out that relatively weak brain stimulation was induced in the un-injured hemisphere of our animals since the stimulator coils are relatively large for a rat. Intriguingly, by treating healthy non-stroke rats with rTMS, we demonstrated that vascular dynamics and angiogenic activity in the brain vasculature were impacted only in animals receiving PT stroke. Our study suggests that ischemic insult stimulates angiogenesis in the first place due to signaling from ischemic tissues. Application of rTMS can further stimulate and support this activity, likely by improving the damaging microenvironment induced by stroke in the near-infarct region.

## Conclusion

These results, taken together, demonstrate for the first time that rTMS exhibits powerful protective and restorative properties on the peri-infarct microvasculature, preserving structure, morphology, and function. We found that rTMS beneficially modulated the post-stroke cytokine response and promoted A2-astrocytic association with cerebral microvessels. As well, several factors relating to repair and angiogenesis, including TGFβ, VEGF, IGFBP1, and PDGFRβ, were upregulated in close association with both vessels and their adjacent astrocytes. Finally, we demonstrated that HIF-1α signaling is highly induced by rTMS. We believe that this increase in HIF-1α, alongside the actions of vessel-associated A2 astrocytes, drives the aforementioned signaling pathways and, therefore, the cumulative effects of rTMS, as demonstrated in Figure 10C. Further work, however, should examine whether or not this is causative rather than simply an association.

When taken into consideration alongside our previous results, these results position rTMS as a potent therapeutic strategy for ischemic stroke 41. In addition, rTMS may hold promise as an adjunct therapy for the current primary approved treatment for, tPA thrombolysis. rTMS dramatically protected the integrity of existing vasculature and prevented excessive BBB permeabilization. TPA, while extremely valuable, can sometimes result in hemorrhagic transformation that can potentially worsen damage done by stroke, necessitating rapid diagnostic imaging. By protecting the existing vasculature, rTMS may reduce the odds and severity of this potentially lethal adverse event 108. This may also represents a future line of research in applying rTMS to hemorrhagic stroke models. If our results can translate to the clinic, then non-invasive rTMS therapy may promote the recovery of stroke survivors by protecting and repairing the vasculature directly impacted by ischemic insult.

## Abbreviations

rTMS: repetitive Transcranial Magnetic Stimulation
PT: photothrombotic
BBB: blood-brain barrier
VEGF: vascular endothelial growth factor
PDGFRβ: platelet-derived grow factor receptor beta
IGFBPs: insulin-like growth factor binding proteins
DAB: 3,3′-diaminobenzidine
SDS-PAGE: sodium dodecyl sulfate-poly-acrylamide gel electrophoresis
HIF-1α: hypoxia-inducible factor 1α
Col IV: collagen IV
MCAO: middle cerebral artery occlusion
